# Correlation between sagittal and inclined angles of cervical facet joints and cervical disc herniation: a radiological observational study

**DOI:** 10.3389/fsurg.2025.1705681

**Published:** 2026-01-26

**Authors:** Rui Weng, Hao Liu, Dongxin Lin, Haiwei Guo, Ling Mo, Hongjiang Liu, Genfu Zhu, Yikai Li, Caijun Liu, Xuecheng Huang, Zhensong Yao

**Affiliations:** 1 School of Traditional Chinese Medicine, Southern Medical University, Guangzhou, Guangdong, China; 2 The Third Affiliated Hospital of Guangzhou University of Chinese Medicine, Guangzhou, Guangdong, China; 3 Guangdong Research Institute for Orthopedics and Traumatology of Chinese Medicine, Guangzhou, Guangdong, China; 4 The First Affiliated Hospital of Guangzhou University of Chinese Medicine, Guangzhou, Guangdong, China; 5 School of Medicine, Fuzhou University, Fuzhou, Fujian, China; 6 School of Basic Medicine, Southern Medical University, Guangzhou, Guangdong, China; 7 Shenzhen Hospital (Futian) of Guangzhou University of Chinese Medicine, Shenzhen, Guangdong, China; 8 Baiyun Hospital of the First Affiliated Hospital of Guangzhou University of Chinese Medicine, Guangzhou, Guangdong, China

**Keywords:** asymmetry, cervical disc herniation, cervical facet joints, inclined angle, sagittal angle

## Abstract

**Background:**

Previous studies have found that asymmetry of cervical facet joints is associated with cervical disc herniation. However, the effect of facet joint angle on disc herniation is inconclusive. Further identification of the pathological anatomic features of cervical disc herniation is helpful for the prevention and treatment of the disease.

**Objective:**

To explore the relationship between sagittal angle or inclined angle of cervical facet joint and CDH.

**Methods:**

Among patients who visited the First Affiliated Hospital and the Third Affiliated Hospital of Guangzhou University of Chinese Medicine from June 2015 to December 2022, 271 patients with single-segment CDH (79 in C4/5 segment, 122 in C5/6 segment, and 70 in C6/7 segment) were screened for inclusion in the CDH group. At the same time, 132 age- and gender-matched healthy subjects were randomly enrolled as a control group. Data on the bilateral sagittal angles and inclined angles of cervical facet joints were collected from both groups. Intergroup comparisons were performed after Bonferroni correction and adjustment for confounding factors.

**Results:**

There were no significant differences in gender, age, or BMI between the two groups. However, the C2–7 Cobb angle was significantly smaller and the intervertebral disc height at the corresponding affected segments was significantly lower in the CDH group than in the control group (*P* < 0.05). Regarding the sagittal angle of facet joints: before correction, the bilateral differences and asymmetry rates of sagittal angles at all segments were significantly higher in the CDH group than in the control group (*P* < 0.05); after Bonferroni correction, only the difference at the C4/5 segment remained significant (*P* < 0.05). The average sagittal angle at the C5/6 segment was significantly higher in the CDH group than in the control group before correction (*P* < 0.05), but this difference disappeared after adjusting for confounding factors. Regarding the inclined angle of facet joints: before correction, the bilateral differences and asymmetry rates of inclined angles at all segments were significantly higher in the CDH group than in the control group (*P* < 0.05), but no significant differences were observed after correction. The average inclined angles at the C5/6 and C6/7 segments were significantly lower in the CDH group (*P* < 0.05), and these differences persisted after adjusting for confounding factors (*P* < 0.05).

**Conclusion:**

Sagittal angle asymmetry of facet joints at the C4/5 segment is associated with lower cervical disc herniation. A significant reduction in the average inclined angle (tendency to be horizontal) of the facet joints at the corresponding segments in patients with single-segment CDH at C5/6 and C6/7 is an important pathophysiological and anatomical characteristic of the disease. Additionally, the intergroup difference in the average sagittal angle may be influenced by the degree of cervical lordosis and intervertebral disc height.

## 1 Introduction

With the aging of the population and changes in work styles caused by the development of modern society, the incidence of cervical disc herniation (CDH) has gradually increased and is affecting younger individuals (1), which has caused a huge burden to health expenditure (2, 3). Treatment of CDH mainly involves conservative and surgical treatment. Conservative treatment targets local inflammation, while surgery is indicated for severe cases or when conservative measures fail, aiming to relieve nerve root or spinal cord compression (3). Identifying the factors of CDH will not only help prevent degenerative cervical spine disease, but also help further guide treatment plans.

The triple-articular complex, consisting of the intervertebral disc and bilateral facet joints, plays a vital biomechanical role in providing spinal mobility and restricting excessive movement (4). Any abnormality in one joint can affect the others. It has been reported that intervertebral disc degeneration can aggravate degeneration of facet joints and osteoarthritis (5). On the other hand, asymmetry of facet joint, defined as asymmetries in the direction of the facet joints on the left and right sides (6), had also often been reported to be associated with degenerative disc diseases (6–11). Xu et al. (6) found that sagittal angle asymmetry of cervical facet joints was more prevalent in patients with degenerative cervical spondylolisthesis. In other studies, it has also been found that cervical facet joint asymmetry is correlated with CDH (10, 11). In addition, the finite element research found that sagittal angle asymmetry of cervical facet joint may be an anatomic risk factor for cervical disc or facet joint degeneration (12).

Despite extensive research, the role of the sagittal angle of cervical facet joints in disc herniation remains controversial. Wang et al. (11) reported that the magnitude of the left or right facet joint sagittal angle did not influence the direction of disc herniation. In contrast, Huang et al. (10) demonstrated that the side with a larger sagittal angle was more prone to disc herniation, attributing this to reduced resistance against cervical axial rotation, which increases intervertebral disc stress. A subsequent finite element analysis (13) supported the latter finding, showing that larger sagittal angles correlate with increased spinal motion and disc stress, thus elevating the risk of herniation in asymmetric cervical segments. However, there is a critical gap in current research: previous studies have mostly taken facet joint sagittal angle asymmetry as the core research variable, while the independent impact of the absolute value of sagittal angle on cervical disc herniation (CDH) has not been fully explored. This research gap prevents us from clarifying whether the inherent anatomical characteristics of facet joints (rather than merely the relative differences between the two sides) increase the risk of disc herniation. Therefore, this study will focus on the analysis of the absolute value of sagittal angle to fill this gap, thereby breaking through the previous research framework centered on asymmetry and more directly revealing the impact of the inherent geometric characteristics of facet joints on the pathogenesis of CDH. In addition, previous research has shown that cervical facet joints can be characterized by their sagittal angle in the horizontal plane and an inclined angle (i.e., facet orientation angle) in the sagittal plane (14). During development from birth to adulthood, the inclined angle becomes more vertical, enhancing cervical spinal stability (14). Conversely, a more horizontal inclined angle has been associated with cervical spondylolisthesis and degeneration (15, 16). This is due to the reduced resistance to vertebral body sliding with a horizontal—oriented inclined angle, which compromises spinal stability and increases the risk of intervertebral disc injury and degeneration (17).

Based on the aforementioned research gaps and biomechanical principles, this study proposes the hypothesis that compared with healthy controls, patients with CDH tend to exhibit larger absolute values of facet joint sagittal angles and more horizontal inclined angles. Compared with previous studies focusing on asymmetry, evaluating absolute angle values provides a novel biomechanical perspective for two main reasons: Firstly, absolute angle values directly reflect the inherent structural and geometric characteristics of facet joints, which are crucial determinants of their core biomechanical functions (such as load-bearing capacity and motion restriction) and are not affected by bilateral symmetry. For instance, even in the absence of bilateral facet joint asymmetry, a segment with a large absolute sagittal angle may inherently lack sufficient resistance to cervical axial rotation, thereby increasing intervertebral disc stress and the risk of herniation. Secondly, focusing on absolute values avoids confounding by bilateral compensatory effects: bilateral facet joint asymmetry may mask the true impact of individual facet joint geometric features, whereas absolute value analysis can isolate the independent structural contribution of each facet joint to spinal stability. This research approach helps clarify how the inherent anatomical characteristics of facet joints increase susceptibility to CDH, complementing and extending previous findings (10, 11). To test the above hypothesis, this study is a retrospective multicenter case-control study focusing on the lower cervical segments (C4/5, C5/6, C6/7) to explore the association between cervical facet joint sagittal angles, inclined angles, and single-segment CDH. By analyzing differences in these absolute angle values between the two groups, we aim to clarify their role in the pathogenesis of CDH and provide new insights for the prevention and treatment of this disease.

## 2 Materials and methods

### 2.1 Overview of the CDH group

Among the patients treated in the First and Third Affiliated Hospitals of Guangzhou University of Chinese Medicine from June 2015 to December 2022, 271 patients with cervical spondylotic radiculopathy caused by single-segment CDH were included in the CDH group, including 123 males and 148 females, with a mean age of 48.79 ± 12.74 years old (21–66 years old). This study was a retrospective multicenter case-control study.

### 2.2 Inclusion criteria of the CDH group

Patients with neck pain or discomfort and upper limb pain due to CDH were included in the CDH group. Detailed inclusion criteria include:

1. Patients with single-level CDH in C4/5, C5/6 or C6/7, and they underwent either conservative or operative treatment;
2. Symptoms were mainly typical radicular symptoms such as upper limb pain, numbness, and/or neck pain;
3. At least 1 of the 3 orthopedic examinations for cervical spondylotic radiculopathy caused by cervical disc herniation showed positive signs: (1) Spurling test; (2) Cervical distraction test; (3) Upper limb tension test.
4. The clinical signs and symptoms were consistent with CT and MRI of the cervical spine.

### 2.3 Overview and inclusion criteria of the control group

A total of 132 persons without cervical spine disease matching the age and gender of the herniation group were found in the CT database as the control group, including 70 males and 62 females, with an average age of 48.14 ± 15.68 years old. These participants were healthy people who underwent the physical examination and underwent cervical CT. The radiologists and spine surgeon in charge of enrollment would confirm that the included control group did not have disc herniations.

### 2.4 Exclusion criteria of the CDH group and the control group

1. Those combined with cervical spondylotic myelopathy, vertebral artery cervical spondylosis, sympathetic cervical spondylosis, esophageal cervical spondylosis or mixed cervical spondylosis;
2. Those with ossification of posterior longitudinal ligament or cervical spondylolisthesis;
3. Those with fused vertebrae or other deformities in the cervical spine, or severe osteoporosis;
4. Those with severe trauma, infection, cervical spine tumors, tuberculosis and other bone diseases in the cervical spine and neck soft tissue;
5. Those with a history of cervical spine surgery;
6. Those with incomplete information or unclear images.

### 2.5 Parameters of cervical CT and MRI scan

The CT scan used a 64-slice spiral CT scanner (SOMATOM Definition AS+, Siemens, Germany). The detailed CT parameters are as follows: tube voltage 120–140 kV, tube current 140–280 mA, rotation time 0.75 s, matrix 512 × 512, field of view: 350 mm, layer thickness 1.25 mm, interval 0.625 mm, bone window reconstruction, conventional soft tissue window.

The MRI scan was performed with MAGNETOM Verio 3.0T (Siemens, Germany). Scanning parameters of sagittal T2WI images were as follows: repetition time/echo time/number of excitation (TR/TE/NEX) were 2,000 ms/100 ms/4, slice thickness/slice spacing(ST/SP) were 2.0 mm/0.5 mm, and the number of scanning layers (Num) was 12. Scanning parameters of sagittal T1-FLAIR image were as follows: TR/TE/NEX were 2700 ms/25 ms/2, ST/SP were 2.0 mm/0.5 mm, and the Num was 12. Scanning parameters of axial T2WI images were as follows: TR/TE/NEX were 1,800 ms/120 ms/4, ST/SP were 1.5 mm/0.5 mm, and the Num was 15.

### 2.6 Measurement method of the sagittal angle and inclined angle of cervical facet joint

**Figure 1:**
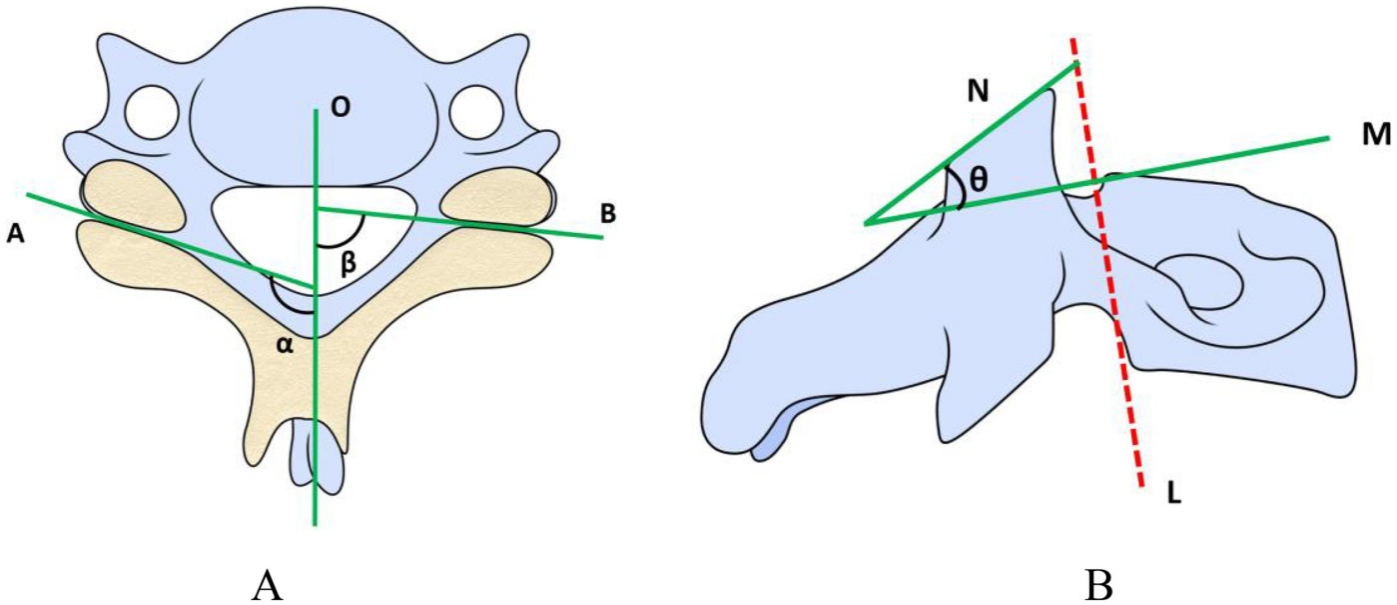
**(A)** schematic diagram of the method for measuring the sagittal angle of the facet joint. **(B)** Schematic diagram of the method for measuring the inclined angle of the facet joint.

In this study, referring to the method of Xu et al. (6), the sagittal angle of the cervical facet joint was measured on axial CT. In the measurement, the cross-section of axial CT of the cervical spine was parallel to the lower endplate of the upper vertebra. A median sagittal line was drawn from the midpoint of the intervertebral disc to the midpoint of the base of the cervical spinous process, and the facet joint line was drawn on both sides parallel to the upper and lower facet joint spaces. In this way, the angle between the median sagittal line and the facet joint line is the sagittal angle of the cervical facet joint (Figure 1A, *α*&*β*).

The measurement of inclined angle of cervical facet joint (i.e., facet orientation angle) was based on the method of Pesenti et al. (14). The inclined angle of cervical facet joint was measured on the sagittal plane. First, draw a parallel straight line along the posterior wall of the vertebral body (Figures 1B,L), and draw the perpendicular line M to the straight line L. In this way, the angle between line N and line M is the inclined angle of the cervical facet joint on that side (Figure 1B, *θ*).

According to the standard of Rong et al. (12), the sagittal angle/inclined angle difference of more than 7° between the left and right facet joints of the cervical spine was defined as sagittal angle/inclined angle asymmetry of cervical facet joints. The CT images of the cervical spine were obtained and measured in the imaging system by two spine surgeons with at least 3 years of clinical experience, who independently and randomly performed single-blind evaluations (neither the patient's medical record information nor the original imaging diagnosis information was provided). In order to reduce the measurement error, the average value of the measurement is used as the final measurement value.

### 2.7 Statistical analysis

SPSS13.0 software (SPSS, Chicago, Illinois, USA) was used for statistical analysis, and the measurement data were statistically described using mean ± standard deviation. The Kolmogorov–Smirnov test and Shapiro–Wilk test were used to verify the normality of the data, and the homogeneity of variance test was applied to assess the consistency of variances: If the data were normally distributed and homogeneous in variance, the independent-sample t test was adopted, with the mean difference (MD) and its 95% confidence interval (95% CI) reported, and the effect size Cohen's d calculated. If the above conditions were not met, the nonparametric test (Mann–Whitney U test) was used, with the Z value reported and the effect size r calculated using the formula *r* = |Z|/√N (where N is the total sample size included in the comparison of the corresponding segment). Intergroup differences in categorical variables were compared using the Pearson Chi-square test. Categorical variables were expressed as counts, and intergroup comparisons were performed using the *χ*^2^ test; for binary outcomes such as “asymmetry status”, the odds ratio (OR) and its 95% CI were reported.

Comparisons were conducted separately by cervical segment (C4/5, C5/6, C6/7). Bonferroni correction was applied for multiple comparisons of the same indicator across the three segments (correction formula: Padj = P × 3; adjusted significance level *α* = 0.05/3). To control for confounding factors, multiple linear regression analysis was further performed for continuous primary outcomes (e.g., average sagittal angle and average inclined angle of bilateral facet joints): the average value of bilateral angles was taken as the dependent variable, group (CDH vs. control) as the primary independent variable, and age, body mass index (BMI), C2–7 Cobb angle, and disc height of the corresponding segment as covariates. The adjusted regression coefficient (β), 95% CI, and *P* value were reported. The statistical significance level was set at *P* < 0.05; for comparisons involving Bonferroni correction, the adjusted *P* value (Padj) and adjusted α were used as the criteria.

## 3 Results

A total of 271 patients with CDH were included in the CDH group, including 79 patients with C4/5 CDH, 122 patients with C5/6 CDH, and 70 patients with C6/7 CDH. All of the cases were posterior herniation of the intervertebral disc, with left side herniation in 76 cases, right side herniation in 63 cases and central type herniation in 132 cases. The control group included 132 healthy subjects, with measurements performed separately on the C4/5, C5/6, and C6/7 segments, resulting in a total of 396 pairs of facet joint CT data. The CT scanning equipment, scanning parameters, and measurement methods were consistent between the control group and the CDH group.

Baseline data of the two groups were presented in Table 1. There were no statistically significant differences in gender, age, or body mass index (BMI) between the two groups (all *P* > 0.05), indicating overall comparability; however, the C2–7 Cobb angle in the CDH group was significantly smaller than that in the control group. In addition, the disc height of the corresponding affected segments in the CDH group was significantly different from that of the same segments in the control group. Therefore, the C2–7 Cobb angle and the disc height of the corresponding segments were adjusted as confounding factors in the subsequent analyses.

**Table 1:** Comparison of baseline data between the CDH group and control group.

Variable	CDH group (*n* = 271)	Control group (*n* = 132)	Test statistic	P value
Gender (n, Male/Female)	123/148	70/62	*χ*² = 2.078	0.149
Age (Years, mean ± SD)	48.79 ± 12.74	48.14 ± 15.68	*t* = 0.414	0.679
BMI (kg/m², mean ± SD)	22.43 ± 2.89	22.54 ± 2.49	*t* = −0.534	0.594
C2–7 Cobb angle (°, mean ± SD)	6.79 ± 9.62	13.58 ± 11.22	*t* = −5.967	0.000
Disc height (mm, mean ± SD)				
C4/5	3.06 ± 0.69	3.30 ± 0.75	*t* = −2.394	0.018
C5/6	2.79 ± 0.66	3.22 ± 0.74	*t* = −5.015	0.000
C6/7	2.84 ± 0.73	3.32 ± 0.74	*t* = −4.513	0.000

1. Statistical methods: Pearson's chi-square test was used for gender; independent samples *t*-test was applied for age, BMI, C2–7 Cobb angle, and intervertebral disc height. 2. Definition of C2–7 Cobb angle: the angle between the lines connecting the inferior endplates of C2 and C7, reflecting the degree of global cervical lordosis. 3. Intervertebral disc height: the average value of the heights of the anterior, middle, and posterior margins of the intervertebral disc at each segment.

### 3.1 Comparison of sagittal angle differences of facet joints on both sides of cervical spine

The comparison results of the bilateral sagittal angle differences of facet joints between the CDH group and the control group were shown in Table 2. Before correction, the bilateral sagittal angle differences of the CDH group at the C4/5, C5/6, and C6/7 segments were statistically significantly different from those of the control group (*P* values = 0.010, 0.028, and 0.046, respectively). After Bonferroni correction (number of corrections = 3, corresponding to multiple comparisons of 3 cervical segments), only the sagittal angle difference at the C4/5 segment in the CDH group was significantly higher than that in the control group (adjusted *P* = 0.030) with a small effect size (*r* = 0.18, 95% CI: 0.04–0.32). Although the differences at the C5/6 and C6/7 segments showed an increasing trend, no statistically significant differences were observed after correction (adjusted *P* = 0.084 and 0.138, respectively), and both had small effect sizes (*r* = 0.15 and 0.14, with 95% CIs of 0.01–0.29 and 0.00–0.28, respectively).

**Table 2:** Comparison of bilateral sagittal facet joint angle difference between the CDH group and the control group.

Segment	CDH group (°)	Control group (°)	Z-Value	*P*-Value	*P*-Value (Adjusted)	Effect size (*r*)
C4/5	8.46 ± 6.62	5.79 ± 4.37	−2.578	0.010	0.030	0.18 (0.04–0.32)
C5/6	7.98 ± 6.16	5.93 ± 4.19	−2.201	0.028	0.084	0.15 (0.01–0.29)
C6/7	6.99 ± 4.75	5.63 ± 4.34	−1.999	0.046	0.138	0.14 (0.00–0.28)

1. The Wilcoxon rank-sum test was used for intergroup comparisons, with Z as the test statistic. 2. Bonferroni correction was applied for multiple comparisons across 3 segments, and a Adjusted *P* < 0.05 was considered statistically significant. 3. Criteria for effect size classification: *r* = 0.1–0.3 indicates a small effect, 0.3–0.5 a medium effect, and > 0.5 a large effect.

Asymmetry of the sagittal angle was defined as a bilateral difference in the sagittal angles of cervical facet joints >7°. Further comparisons of the number of asymmetric cases at each segment between the two groups were shown in Table 3. Before correction, the proportions of sagittal angle asymmetry at the C4/5, C5/6, and C6/7 segments in the CDH group were significantly higher than those in the control group (*P* values = 0.011, 0.021, and 0.026, respectively). After Bonferroni correction, only the number of asymmetric cases at the C4/5 segment in the CDH group was significantly higher than that in the control group (adjusted *P* = 0.033, OR = 2.09, 95% CI: 1.18–3.69). Although the proportions of asymmetry at the C5/6 and C6/7 segments showed an increasing trend, no statistically significant differences were observed after correction (adjusted *P* = 0.063 and 0.078, respectively).

**Table 3:** Comparison of the number of cases of sagittal angle asymmetry of the facet joints between the CDH group and the control group.

Segment	Group	Cases (*n*)	Whether it is asymmetrical?		OR (95% CI)	*χ*^2^-value	*P*-value	*P*-value (Adjusted)
			Yes (*n*)	No (*n*)				
C4/5	CDH group	79	41	38	2.09 (1.18–3.69)	6.491	0.011	0.033
	Control group	132	45	87				
C5/6	CDH group	122	55	67	1.82 (1.09–3.04)	5.302	0.021	0.063
	Control group	132	41	91				
C6/7	CDH group	70	34	36	1.95 (1.08–3.54)	4.962	0.026	0.078
	Control group	132	43	89				

1. Intergroup comparisons were performed using the Pearson chi-square test, with *χ*^2^ as the test statistic. 2. Bonferroni correction was applied for multiple comparisons across 3 segments, and an adjusted *P* < 0.05 was considered statistically significant.

### 3.2 Comparison of average sagittal angle of facet joints on both sides of cervical spine

The comparison results of the average bilateral sagittal angles of facet joints between the CDH group and the control group were shown in Table 4. Before correction, only the average sagittal angle at the C5/6 segment in the CDH group was statistically significantly different from that in the control group (*P* = 0.008), while no significant differences were observed at the C4/5 and C6/7 segments (*P* = 0.104 and 0.494, respectively). After Bonferroni correction, the average sagittal angle at the C5/6 segment in the CDH group remained significantly higher than that in the control group (adjusted *P* = 0.024, effect size *r* = 0.166); no significant differences were found at the C4/5 and C6/7 segments after correction either (adjusted *P* = 0.312 and 1.000, respectively).

**Table 4:** Comparison of average sagittal angle of facet joints on both sides between the CDH group and the control group.

Segment	CDH group(°)	Control group(°)	Z-value	*P*-value	*P*-value (Adjusted)	Effect size (*r*)
C4/5	92.36 ± 7.58	90.66 ± 8.53	−1.627	0.104	0.312	0.112
C5/6	96.29 ± 7.92	93.32 ± 8.07	−2.653	0.008	0.024	0.166
C6/7	92.35 ± 7.36	90.89 ± 7.89	−0.684	0.494	1.000	0.048

1. Bonferroni correction was applied for multiple comparisons across 3 segments, and an adjusted *P* < 0.05 was considered statistically significant. 2. Criteria for effect size classification: an r value of 0.1–0.3 indicates a small effect, 0.3–0.5 a medium effect, and >0.5 a large effect.

After further adjusting for the C2–7 Cobb angle and the intervertebral disc height at the corresponding segments, a multiple linear regression analysis was performed on the average bilateral sagittal angles of facet joints at each segment (Table 5). With the average bilateral sagittal angle of facet joints as the dependent variable and group (CDH group vs. control group) as the main independent variable, no statistically significant intergroup differences were observed at any segment after Bonferroni correction following adjustment for confounding factors (adjusted *P* = 0.549, 0.105, and 0.570, respectively).

**Table 5:** Multivariable linear regression analysis of segmental average bilateral sagittal angle of the facet joints after adjusting for the C2–7 cobb angle and the corresponding segmental disc height.

Segment	Adjusted model *β*(°)	95% CI	P-Value (Corrected)
C4/5	1.634	−0.777 to 4.044	0.549
C5/6	2.282	0.160 to 4.404	0.105
C6/7	1.648	−0.825 to 4.122	0.570

1. β value: the difference in the average sagittal angle of the CDH group relative to the control group (after adjusting for confounding factors). 2. Bonferroni correction was applied for multiple comparisons across 3 segments, and an adjusted *P* < 0.05 was considered statistically significant.

### 3.3 Comparison of inclined angle difference of facet joints on both sides of cervical spine

The comparison results of the bilateral inclined angle differences of facet joints between the CDH group and the control group were shown in Table 6. Before correction, the bilateral inclined angle differences of the CDH group at the C4/5, C5/6, and C6/7 segments were statistically significantly different from those of the control group (*P* values = 0.034, 0.018, and 0.027, respectively). After Bonferroni correction, no statistically significant differences were observed at the aforementioned segments (adjusted *P* = 0.102, 0.054, and 0.081, respectively), but the effect sizes indicated small effects (r = 0.106, 0.118, and 0.110, respectively).

**Table 6:** Comparison of inclined angle difference of the facet joints on both sides between the CDH group and the control group.

Segment	CDH group (°)	Control group (°)	Z-value	*P*-value	*P*-value (Adjusted)	Effect size (*r*)
C4/5	4.98 ± 3.84	3.73 ± 2.90	−2.120	0.034	0.102	0.106
C5/6	5.43 ± 4.25	4.05 ± 3.09	−2.366	0.018	0.054	0.118
C6/7	5.58 ± 4.08	4.19 ± 3.05	−2.209	0.027	0.081	0.110

1. The Wilcoxon rank-sum test was used for intergroup comparisons, with Z as the test statistic. 2. Bonferroni correction was applied for multiple comparisons across 3 segments, and a Adjusted *P* < 0.05 was considered statistically significant. 3. Criteria for effect size classification: *r* = 0.1–0.3 indicates a small effect, 0.3–0.5 a medium effect, and >0.5 a large effect.

Inclined angle asymmetry was defined as a bilateral difference in the inclined angles of cervical facet joints >7°. Further comparisons of the number of asymmetric cases at each segment between the two groups were presented in Table 7. Before correction, the proportions of inclined angle asymmetry at the C4/5, C5/6, and C6/7 segments in the CDH group were significantly higher than those in the control group (*P* values = 0.020, 0.035, and 0.039, respectively). After Bonferroni correction, although the proportions of asymmetry at the aforementioned segments showed an increasing trend, no statistically significant differences were observed (adjusted *P* = 0.060, 0.105, and 0.117, respectively).

**Table 7:** Comparison of the number of cases of inclined angle asymmetry of facet joint between the CDH group and the control group.

Segment	Group	Cases (*n*)	Whether it is asymmetrical?		OR(95% CI)	*χ*^2^-value	*P*-value	*P*-value (Adjusted)
			Yes (*n*)	No (*n*)				
C4/5	CDH group	79	24	55	2.18 (1.12–4.23)	5.451	0.020	0.060
	Control group	132	22	110				
C5/6	CDH group	122	37	85	1.86 (1.04–3.33)	4.457	0.035	0.105
	Control group	132	25	107				
C6/7	CDH group	70	23	47	2.00 (1.03–3.85)	4.312	0.039	0.117
	Control group	132	26	106				

1. Intergroup comparisons were performed using the Pearson chi-square test, with χ² as the test statistic. 2. Bonferroni correction was applied for multiple comparisons across 3 segments, and an adjusted *P* < 0.05 was considered statistically significant.

### 3.4 Comparison of average inclined angle of facet joints on both sides of cervical spine

The comparison results of the average bilateral inclined angles of facet joints between the CDH group and the control group were presented in Table 8. Before correction, the average inclined angles at the C5/6 and C6/7 segments in the CDH group were statistically significantly different from those in the control group (*P* = 0.003 and 0.000, respectively), while no significant difference was observed at the C4/5 segment (*P* = 0.054). After Bonferroni correction, the average inclined angles at the C5/6 and C6/7 segments in the CDH group remained significantly lower than those in the control group (adjusted *P* = 0.009 and 0.000, respectively). The effect sizes indicated a small effect at the C5/6 segment (*r* = 0.189) and a medium effect at the C6/7 segment (*r* = 0.319); no significant difference was found at the C4/5 segment after correction either (adjusted *P* = 0.162, *r* = 0.132).

**Table 8:** Comparison of average inclined angle of facet joints on both sides between the CDH group and the control group.

Segment	CDH group(°)	Control group(°)	Z-value	*P*-value	*P*-value (Adjusted)	Effect size (*r*)
C4/5	43.65 ± 6.27	45.49 ± 5.73	−1.923	0.054	0.162	0.132
C5/6	45.42 ± 6.63	47.88 ± 5.87	−3.004	0.003	0.009	0.189
C6/7	54.43 ± 6.67	58.67 ± 5.77	−4.530	0.000	0.000	0.319

1. Bonferroni correction was applied for multiple comparisons across 3 segments, and an adjusted *P* < 0.05 was considered statistically significant. 2. Criteria for effect size classification: an r value of 0.1–0.3 indicates a small effect, 0.3–0.5 a medium effect, and >0.5 a large effect.

After further adjusting for the C2–7 Cobb angle and the intervertebral disc height at the corresponding segments, a multiple linear regression analysis was performed on the average bilateral inclined angles of facet joints at each segment (Table 9). With the average bilateral inclined angle of facet joints as the dependent variable and group (CDH group vs. control group) as the main independent variable, after adjusting for confounding factors, the average inclined angles at the C5/6 and C6/7 segments in the CDH group were significantly lower than those in the control group (adjusted *P* = 0.000 and 0.000, with adjusted *β* = −3.780° and −4.580°, respectively); the C4/5 segment did not reach statistical significance after correction (adjusted *P* = 0.063, adjusted *β* = −2.006°).

**Table 9:** Multivariable linear regression analysis of the segmental average bilateral inclined angle of the facet joints after adjusting for the C2–7 cobb angle and the corresponding segmental disc height.

Segment	Adjusted model β(°)	95% CI	P-Value (Corrected)
C4/5	−2.006	−3.713 to −0.299	0.063
C5/6	−3.780	−5.498 to −2.062	0.000
C6/7	−4.580	−6.502 to −2.659	0.000

1. β value: The adjusted difference in the average inclined angle between the CDH group and the control group; a negative β value indicates a lower average inclined angle in the CDH group. 2. Bonferroni correction was applied for multiple comparisons across 3 segments, and an adjusted *P* < 0.05 was considered statistically significant.

## 4 Discussion

The facet joints of the spine are bony contacts between two vertebral bodies that guide the relative motion of one vertebral body over the other (mobility), while also providing torsional stiffness and resistance to shear (stability) (18). Facet joints are not only different in different individuals, but also vary in different segments of the same individual, and may even be asymmetrical on both sides of the same segment (17). Many previous studies have proved that the orientation of facet joints is related to lumbar disc herniation, lumbar spondylolisthesis, and lumbar spinal stenosis (19, 20). However, relatively few studies have been conducted on the cervical spine. Previous studies mainly focused on the relationship between sagittal angle asymmetry of cervical facet joints and cervical degenerative diseases. They concluded that sagittal angle asymmetry of cervical facet joints is related to CDH and cervical spondylolisthesis (6, 9–11). In addition, some studies have found that the more horizontal the cervical facet joint (that is, the smaller the inclined angle of the cervical facet joint) is associated with cervical spondylolisthesis and cervical spinal cord injury (15, 16, 21). However, few studies have conducted on the relationship between the size of the sagittal angle or inclined angle of the cervical facet joint and CDH.

This study focused on comparing patients with single-segment CDH and healthy subjects, excluding cases of multi-segment herniation. The primary rationale is that the pathogenesis of multi-segment CDH is more complex, potentially involving the superimposition of multiple factors such as multi-segment facet joint degeneration and spinal alignment abnormalities. Furthermore, the biomechanical interactions between different herniated segments are difficult to disentangle, which would confound the analysis of the correlation between facet joint angles and disc herniation in single segments. The C4/5, C5/6, and C6/7 segments are the most common sites for cervical disc herniation, accounting for over 80% of all clinical cases (22), and the association between lesions in these segments and abnormalities in facet joint biomechanical function is more pronounced. In contrast, the upper cervical spine has a unique anatomical structure, primarily responsible for head support and rotation (23). Its facet joint morphology and biomechanical characteristics differ significantly from those of the lower cervical spine, and the clinical incidence of upper cervical disc herniation is relatively low. Previous studies have reported that the orientation of cervical facet joints changes with age both in childhood and adulthood (24, 25). Therefore, the control group in this study was matched by age and gender to patients with single-segment CDH. Referring to the criteria proposed by Rong et al. (12), sagittal angle/inclined angle asymmetry of cervical facet joints was defined as a bilateral difference >7°. Meanwhile, comparisons were conducted between the “proportion of asymmetric cases” and the “average bilateral angle difference” to more comprehensively characterize differences in facet joint morphology. The results of this study demonstrated that before adjustment, the bilateral differences and asymmetry rates of sagittal angles and inclined angles at the C4/5, C5/6, and C6/7 segments in the CDH group were significantly higher than those in the control group, which is consistent with the findings of Huang et al. (10) and Wang et al. (11). Even without applying an explicit asymmetry criterion, the bilateral angle differences in the CDH group remained larger, suggesting that the orientation of bilateral facet joints is more irregular in CDH patients. The biomechanical mechanism underlying this phenomenon may be analogous to that in the lumbar spine: as the only osseous contact structure between adjacent vertebrae, irregular facet joint morphology may lead to uneven stress distribution within the intervertebral disc, thereby promoting disc degeneration and even herniation (7, 12). However, it should be noted that after Bonferroni correction, only the sagittal angle asymmetry at the C4/5 segment remained statistically significant, while the intergroup difference in inclined angle asymmetry vanished. This indicates that the association between sagittal angle asymmetry and CDH may be more prominent at the C4/5 segment, and the impact of inclined angle asymmetry may be modulated by multiple factors, requiring cautious interpretation.

This study also compared the average sagittal angles and average inclined angles of bilateral cervical facet joints between the CDH group and the control group. The results showed that the average sagittal angle of facet joints at the C5/6 segment in the CDH group was significantly higher than that in the control group before adjustment. However, this difference disappeared after adjusting for confounding factors such as the C2–7 Cobb angle and intervertebral disc height, suggesting that the intergroup difference in the average sagittal angle may be influenced by factors including the degree of cervical lordosis and intervertebral disc height, and is not an independently associated feature of CDH. In contrast, the average inclined angles at the C5/6 and C6/7 segments were significantly lower in the CDH group, and these differences persisted after adjusting for confounding factors; the C4/5 segment showed a marginal difference (*P* = 0.063), indicating that the facet joint inclined angles at the herniated segments in CDH patients tend to be more horizontal. Previous studies have reported that the mechanical factors potentially contributing to CDH include not only axial compression but also flexion and torsion during cervical spine movement (26–28). The stress generated during cervical movement is jointly borne by the intervertebral disc and bilateral facet joints. Facet joints can restrict excessive cervical movement and provide shear resistance, which to a certain extent prevents the cervical intervertebral disc from bearing excessive stress (13, 18). Some studies have suggested that a larger sagittal angle of facet joints reduces their resistance to cervical movement and shear force (12, 13), leading to increased cervical instability and greater stress on the intervertebral disc (13). Additionally, a more horizontal inclined angle of facet joints lowers their ability to resist vertebral sliding, which is more likely to affect cervical stability and thereby increase the risk of intervertebral disc injury and degeneration (15, 16). Therefore, the results of this study indicate an association between more horizontal cervical facet joint inclined angles and CDH, but a direct causal relationship cannot be confirmed at present, and this issue requires further research validation. Meanwhile, the sagittal angle lacks independent influence on CDH, and its role may need to be comprehensively judged in combination with other anatomical or functional indicators. This is inconsistent with the conclusions of some previous studies that “an increased sagittal angle impairs shear resistance” (12, 13), which may be related to the adjustment of confounding factors included in this study.

In recent years, minimally invasive spinal surgeries (e.g., keyhole fenestration, endoscopic surgery) (29) have been increasingly applied in the treatment of CDH due to their advantages of minimal invasiveness and rapid recovery. Meanwhile, non-fusion techniques such as cervical disc arthroplasty (CDA) have emerged as promising options (30). Previous studies have reported that the range of motion at the treated segment increases after CDA (31, 32). Therefore, for patients with significantly reduced average facet joint inclined angles (tendency to be horizontal) or sagittal angle asymmetry, potential abnormalities in preoperative cervical biomechanical stability already exist, and more rigorous preoperative evaluation may be required for non-fusion surgeries. This is because facet joints are critical structures for maintaining cervical biomechanical balance, and their morphological abnormalities may impair segmental stability (15, 16). Since non-fusion surgeries do not address facet joint morphology, they may fail to fully correct the underlying mechanical imbalance (30). On the other hand, Tabanli et al. (33) compared the postoperative outcomes between CDA and anterior cervical discectomy and fusion and found that preoperative facet joint orientation and segmental biomechanical characteristics may influence the evolution of postoperative range of motion and the risk of heterotopic ossification. This suggests a potential association between facet joint morphology and the prognosis of non-fusion surgeries. It should be emphasized that this study did not provide direct clinical data on non-fusion surgery failure or suboptimal outcomes in such patients. The above viewpoints are merely inferences based on existing biomechanical mechanisms and relevant clinical studies (33), which require validation in targeted clinical trials in the future. In addition, patients with the aforementioned facet joint characteristics should focus on cervical motion protection during rehabilitation management. Excessive flexion-extension or high-intensity massage may increase the mechanical load on intervertebral discs and nerve roots (21, 34, 35). Therefore, a gentle rehabilitation program tailored to individual conditions is recommended, but this is not an absolute contraindication and should be comprehensively judged in combination with clinical symptoms. It is important to note in clinical practice that radiologically confirmed CDH is not always consistent with patients’ clinical manifestations. Some patients exhibit radiological signs of herniation but no obvious symptoms, while some non-CDH individuals may experience neck pain or limited mobility due to other etiologies (36). Thus, facet joint morphological features (e.g., reduced inclined angles at the C5/6 and C6/7 segments, sagittal angle asymmetry at the C4/5 segment) can serve as CDH-related pathophysiological reference indicators, but they cannot be used alone as the basis for surgical decision-making or treatment contraindications. Individualized judgment should integrate patients’ symptoms, physical signs, comprehensive radiological findings, and preoperative biomechanical assessments. Future studies may further incorporate outcome indicators such as postoperative kinematics and heterotopic ossification incidence (33) to clarify the impact of facet joint morphology on the prognosis of different surgical approaches, thereby providing more direct evidence for precise clinical treatment.

This study has several limitations that warrant further refinement in future research. Firstly, this study only confirmed an association between cervical facet joint-related morphological features and CDH, but the causal relationship between them remains unclear, which requires further verification through prospective study designs. Secondly, the study population was limited to patients with single-segment herniation at the C4/5, C5/6, and C6/7 segments—common sites for CDH—and did not include cases involving other cervical segments, leading to certain limitations in the generalizability of the results. Thirdly, this study only measured and analyzed facet joint angles in the horizontal and sagittal planes, without involving coronal plane angles; the potential impact of angles in this direction on CDH requires further exploration. Finally, potential confounding factors that may affect the cervical mechanical environment, such as occupation type and daily cervical posture, were not fully incorporated into the study, precluding a more comprehensive multifactorial analysis. Based on the aforementioned limitations, future studies may further expand the scope of study participants, integrate multiple potential influencing factors, and conduct more systematic and comprehensive analyses to enhance the scientific rigor and clinical applicability of the research conclusions.

## 5 Conclusion

This study demonstrates that sagittal angle asymmetry of cervical facet joints at the C4/5 segment is associated with lower CDH. Additionally, a significant reduction in the average inclined angle of facet joints (tendency to be horizontal) at the C5/6 and C6/7 segments in CDH patients constitutes an important pathophysiological feature of the disease. Notably, the intergroup difference in the average sagittal angle of facet joints is modulated by confounding factors such as the degree of cervical lordosis and intervertebral disc height, and thus is not an independent correlate of CDH. In conclusion, specific morphological abnormalities of cervical facet joints are closely associated with lower CDH, but the causal relationship between them requires further verification through subsequent prospective studies.

## Data Availability

The original contributions presented in the study are included in the article/Supplementary Material, further inquiries can be directed to the corresponding authors.
